# Fluid Biomarkers in Alzheimer’s Disease and Other Neurodegenerative Disorders: Toward Integrative Diagnostic Frameworks and Tailored Treatments

**DOI:** 10.3390/diagnostics12040796

**Published:** 2022-03-24

**Authors:** Linda Giampietri, Elisabetta Belli, Maria Francesca Beatino, Sara Giannoni, Giovanni Palermo, Nicole Campese, Gloria Tognoni, Gabriele Siciliano, Roberto Ceravolo, Ciro De Luca, Filippo Baldacci

**Affiliations:** 1 Neurology Unit, Department of Clinical and Experimental Medicine, University of Pisa, 56126 Pisa, Italy; lindagiampietri@gmail.com (L.G.); elisabettabelli92@gmail.com (E.B.); palermo.giovanni85@gmail.com (G.P.); campesenicole@gmail.com (N.C.); gloria.tognoni@med.unipi.it (G.T.); gabriele.siciliano@unipi.it (G.S.); roberto.ceravolo@unipi.it (R.C.); filippo.baldacci@unipi.it (F.B.); 2 Psychiatry Unit, Department of Clinical and Experimental Medicine, University of Pisa, 56126 Pisa, Italy; mariaf.beatino@gmail.com; 3 Neurology Unit, Ospedale San Giuseppe, 50053 Empoli, Italy; giannonisara@gmail.com; 4 Laboratory of Morphology of Neuronal Network and Systems Biology, Department of Mental and Physical Health and Preventive Medicine, University of Campania “Luigi Vanvitelli”, 80138 Napoli, Italy

**Keywords:** precision medicine, diagnostic algorithm, neurodegeneration, clinical application, liquid biomarkers, alternative matrices, protein misfolding amplification assays

## Abstract

The diagnosis of neurodegenerative diseases (NDDs) represents an increasing social burden, with the unsolved issue of disease-modifying therapies (DMTs). The failure of clinical trials treating Alzheimer′s Disease (AD) so far highlighted the need for a different approach in drug design and patient selection. Identifying subjects in the prodromal or early symptomatic phase is critical to slow down neurodegeneration, but the implementation of screening programs with this aim will have an ethical and social aftermath. Novel minimally invasive candidate biomarkers (derived from blood, saliva, olfactory brush) or classical cerebrospinal fluid (CSF) biomarkers have been developed in research settings to stratify patients with NDDs. Misfolded protein accumulation, neuroinflammation, and synaptic loss are the pathophysiological hallmarks detected by these biomarkers to refine diagnosis, prognosis, and target engagement of drugs in clinical trials. We reviewed fluid biomarkers of NDDs, considering their potential role as screening, diagnostic, or prognostic tool, and their present-day use in clinical trials (phase II and III). A special focus will be dedicated to novel techniques for the detection of misfolded proteins. Eventually, an applicative diagnostic algorithm will be proposed to translate the research data in clinical practice and select prodromal or early patients to be enrolled in the appropriate DMTs trials for NDDs.

## 1. Introduction

Neurodegenerative Diseases (NDDs) taken together represent a major burden for society. According to estimates, in 2020, Alzheimer′s Disease (AD) and related dementias alone affected 13 million people in Europe and 59 million worldwide [1].

Now that Disease-Modifying Treatments (DMT) are approaching commercialization, healthcare structures will be asked to screen a large audience of possible beneficiaries and select and monitor the suitable subjects in a time- and cost-effective manner.

The question then is who and when? The failed AD clinical trials have taught us that pharmacological intervention even in moderate disease stages shuts the stable door after the horse has bolted [2]. Other than AD, pathophysiological processes in the whole NDDs spectrum start many years before the onset of clinical symptoms [3,4,5,6]. The only possible therapeutic window seems thus to lie between the asymptomatic and mildly symptomatic phases, where purely clinical diagnosis fails to reach enough accuracy [7,8].

Facing these demands, research groups have developed criteria defining diseases at prodromal or even preclinical phases with the support of biomarkers of the hallmark pathophysiological alterations, starting from AD [9,10,11], to Parkinson Disease (PD) [12,13], and lately Dementia with Lewy Bodies (DLB) [14]. Although the classifications tend to describe NDDs as separate entities, it is emerging that phenotypes, physiopathologic mechanisms, and pathologies partially overlap and not as infrequently as thought before [15,16].

In a perspective shift from research to real life, we should be able to intercept those who already are in the trajectory of the disease among subjects at risk for developing NDDs, and fluid biomarkers may turn out useful for this purpose. Broad panels of biomarkers, reflecting specific pathophysiological mechanisms such as misfolded protein accumulation, neuroinflammation, synaptic dysfunction, neuronal injury, genetic expression and regulation, and others, need to be validated to refine diagnosis, designing of clinical trajectories, monitoring of disease progression, and recruiting subjects eligible for targeted therapy.

At the moment, we have few validated pathophysiological biomarkers for NDDs, covering mostly AD and requiring expensive imaging tools or body fluid invasive sampling [15], totally inadequate for utilization in large populations.

Optimization of screening procedures requires easily implementable tests, that are possibly repeatable, minimally-invasive, and with high negative predictive values (NPV). Obviously, blood represents the most suitable and explored source for this purpose, but alternative matrices are currently under evaluation too, e.g., saliva, retina, olfactory mucosa. Second level assessments may include instead core and novel Cerebrospinal Fluid (CSF) biomarkers, and Protein Misfolding Cyclic Amplification (PMCA) assays as innovative tools to characterize NDDs based on different protein strains (sCJD, but also synucleinopathies, and potentially tauopathies too) [17].

As well as the diagnostic phase, biomarkers will represent an invaluable tool for therapy management. Now-running trials on DMTs take biomarkers into account in variable proportion. They are used for the selection of participants, as outcome measures in terms of target engagement, surrogates for efficacy on downstream pathologic events, and participants stratification.

In this paper, we overviewed the state of the art for the latest fluid biomarkers of AD, Parkinson′s Disease (PD), atypical parkinsonism, Frontotemporal Dementia (FTD), amyotrophic lateral sclerosis (ALS), as major NDDs, addressing their potential use as screening, diagnostic and prognostic tools, and their implementation in trials for drugs in late stages of clinical development, namely phase II and III. A comprehensive and systematic review of all the available literature on fluid biomarkers in major NDDs is an effort that goes beyond the scope of this paper. We focused on the most promising biomarkers in our opinion, both those related to core pathophysiological mechanisms, on blood and alternative matrices, even not properly fluid but easily accessible, and the emerging ones on CSF. Attention was brought in particular to novel ultrasensitive technique and protein misfolding amplification assays, that may turn out useful in the screening process. Finally, unmet needs for fluid biomarkers will be discussed and a diagnostic algorithm will be proposed for biomarker implementation in clinical practice.

## 2. Fluid Biomarkers for NDDs

A significant clinical and pathophysiological variability characterizing AD and other NDDs has been emerging lately. Proteostasis impairment, synaptic dysfunction, neuroinflammation, and neuronal damage are now assumed to synergistically determine the neurodegenerative process, resulting in highly heterogeneous clinical syndromes. Several works report concomitance of different underlying proteinopathies at post-mortem examinations on AD brains [18,19]. Thus, purely clinical-based diagnosis cannot reflect the multifaceted pathophysiological mechanisms of NDDs.

Biomarker-based criteria are essential for a presymptomatic or at least prodromal diagnosis and appropriate enrollment in disease-modifying clinical trials. Central nervous system (CNS)-related fluid biomarkers may prove highly valuable for an unbiased description of NDDs. CSF is the most reliable matrix mirroring brain modifications, but CSF sampling is invasive, expensive, and not suitable for screening purposes. Currently, CSF biomarkers are formally integrated into diagnostic criteria for AD (Aβ_1-42_ peptide, total tau (t-tau), and phosphorylated-tau (p-tau) proteins) and Creutzfeldt–Jakob Disease (CJD) (the detection of prion protein (PrP) scrapie using the real-time quaking-induced conversion assay (RT-QuIC)) [9,20,21,22]. By contrast, several biological conditions (e.g., blood-brain-barrier selective passage, analytes dilution, interference of aspecific proteins) represent a concern when measuring biomarkers in plasma samples. The recent introduction of novel ultrasensitive methods has properly addressed most of these issues. As opposed to traditional enzyme-linked immunosorbent assay (ELISA), broadly applied for CSF analysis but unable to replicate its performance in blood, these techniques display subfemtomolar limits of detection (LoD), leading to a substantial optimization of measurement procedures [17].

## 3. Candidate Biomarkers

In recent years, thanks to the increasing availability of novel detection methods (e.g., single molecule array or Simoa, electrochemiluminescence immunoassays or ECLIA, and immunomagnetic reduction or IMR), NDDs biomarker research has moved towards blood. Great advantages may come from the wide-scale implementation of minimally invasive tests for stratifying large preclinical populations with both diagnostic and therapeutic aims. Growing interest has been addressed to the detection of protein isoforms in bodily fluids. In parallel, biomarkers reflecting neuroinflammation, synaptic dysfunction, and neuronal damage are currently under evaluation. Biomarkers for genetic forms of NDDs will be treated in a specific chapter, together with the associated clinical trials.

### 3.1. Blood Biomarkers

#### 3.1.1. Aβ Peptides

Among subjective memory complainers (SMC) and cognitively unimpaired (CU) subjects, the ratio of Aβ_1-42_ and Aβ_1-40_ determined in blood through the Simoa technique exhibits moderate to good accuracy in correctly predicting the amyloid status as assessed by PET or CSF [23,24,25]. Longitudinal studies carried out with Simoa report an ability of blood Aβ_1-42_ and Aβ_1-42_/Aβ_1-40_ to track AD clinical progression [23,26]. In some investigations involving CU and clinical AD patients, blood Aβ_1-40_ and Aβ_1-42_ ratios assessed through immunoprecipitation coupled with mass-spectrometry (IP-MS) report good to optimal accuracy in differentiating amyloid-positive and negative participants [27,28]. Interestingly, Aβ peptides misfolded species measured with an immunoinfrared sensor (iRS) identify amyloid-PET positivity with moderate accuracy and predict clinical AD diagnosis with optimal accuracy [29,30]. The techniques applied in the studies above have been implemented to accurately quantify Aβ, as this peptide is prone to stick with non-specific molecules through hydrophobic interactions. Simoa represents a fully automated method relying on capturing antibody-coated microbeads that selectively target single Aβ proteins at subfemtomolar concentrations. In IP-MS, a tailored antibody-driven phase is associated with spectrometric analysis after a multi-step procedure aimed at minimizing matrix interference. At last, iRS is designed to quantify the amount of β-sheet enriched conformations of Aβ peptides compared to all Aβ species according to their relative frequency shift in the infrared, prior to a detection step through epitope-specific antibodies.

#### 3.1.2. t-tau

The levels of blood t-tau measured with Simoa in CU subjects are positively related to the risk of conversion to mild cognitive impairment (MCI) [31]. Moreover, a good accuracy of blood t-tau in predicting the tau-PET status among CU, MCI, and AD patients is reported, while this biomarker could poorly discriminate amyloid-positive from negative participants in analogous clinical populations [32,33]. Novel insights come from the measurement of tau protein fragments, the full-length isoform being only a small part of the whole circulating protein pool. In this attempt, good to optimal accuracy was shown by t-tau species detected through N-terminal-directed antibodies in differentiating CU from MCI/AD patients [34]. 

#### 3.1.3. p-tau181

Mounting evidence supports the role of blood p-tau181 as a specific AD biomarker. In a large recent investigation, p-tau181 levels assessed in blood with an ECLIA-based method could identify both clinically- and neuropathologically-defined AD with good to excellent accuracy [35] (Table 1). Most interestingly, a similar performance is displayed by this marker in the discrimination of AD from frontotemporal lobar degeneration (FTLD) with either syndromic or autoptic identification [36,37] (Table 1). Additionally, plasma p-tau181 measured with Simoa differentiates AD from vascular dementia (VaD), Progressive Supranuclear Palsy/Corticobasal Syndrome (PSP/CBS), PD/Multiple System Atrophy (PD/MSA), and behavioral variant of FTD/primary progressive aphasia (bvFTD/PPA) with good to optimal accuracy [37] (Table 1).

#### 3.1.4. p-tau Alternative Isoforms

CSF p-tau217, measured with the traditional ELISA method, performs better than CSF p-tau181 in predicting the Aβ status as assessed by amyloid-PET and in discriminating AD from non-AD dementias [38]. Notably, when determined through N-terminal directed immunoassays, CSF p-tau181 and p-tau217 have a comparable accuracy in the distinction of AD from non-AD clinical pictures [39]. Recent data support the promising role of p-tau217 as a specific indicator of AD pathology when also detected in plasma [40,41] (Table 1). 

Some papers report a higher performance of CSF p-tau231 over p-tau181 in differentiating AD from CU subjects with either a clinical or autoptic confirmation [42]. Intriguingly, in a multicenter study, plasma p-tau231 determined with Simoa shows good to optimal accuracy in properly distinguishing clinical continuum of AD from CU and non-AD dementia subjects. Moreover, an earlier increase of p-tau231 plasma concentration compared to p-tau181 was observed through autoptic examination in AD patients [43] (Table 1). 

#### 3.1.5. Nfl

Although lacking in disease-specificity, blood Nfl is a robust biomarker of axonal degeneration across different NDDs [44]. In particular, Nfl detected in plasma or serum may be exploitable for both diagnostic and prognostic aims in AD [45,46,47,48,49,50] (Table 1). Of note, blood Nfl holds potential as a screening strategy in preclinical AD settings [50,51] (Table 1) and was shown to predict the onset of disease in presymptomatic AD mutation carriers [52,53]. In amyotrophic lateral sclerosis (ALS), significantly higher levels of blood Nfl compared to HC have been largely demonstrated [54,55,56] (Table 1). Solid data suggest a feasible role of serum Nfl as a supportive tool in the diagnostic work-up of ALS and for disease staging as well [54,55,56,57,58,59] (Table 1). As opposed to AD, blood Nfl concentration seems to increase only in the symptomatic stage of ALS mutation carriers [60]. Moreover, Nfl levels in blood may be useful for selectively identifying FTD from psychiatric disorders which often display overlapping clinical pictures [61,62]. Finally, this biomarker seems to be promising for the differential diagnosis between PD and atypical parkinsonisms such as PSP, CBD, and MSA [63,64,65]. Indeed, NfL measured in CSF of patients with pure autonomic failure (PAF)-predicted phenoconversion to MSA was absent in PD and DLB converters and in non-converters [66].

### 3.2. Novel CSF Biomarkers

#### 3.2.1. CSF Ng and Other Synaptic Biomarkers

Synaptic dysfunction and loss are considered early events in the AD pathogenesis and markers tracking these changes are regarded as promising in NDDs [67].

Among proteins reflecting synaptic damage in NDDs, neurogranin (Ng) detected in CSF represents the most convincing biomarker, especially for AD [68]. Higher levels of CSF Ng have been extensively reported in AD patients compared to HC [69,70,71] (Table 1). In several investigations, this protein shows a moderate accuracy in the discrimination between AD and HC [72,73,74] (Table 1). Interestingly, the concentration of CSF Ng was higher in subjects with a typical amnestic phenotype of AD compared to atypical AD cases, suggesting a possible link with a selective hippocampal degeneration [75]. Robust evidence supports the role of CSF Ng as a staging and prognostic biomarker throughout the entire course of AD [69,70,76,77,78]. As for other biomarkers of neurodegeneration, a massive release is likely in the CSF and blood of CDJ patients. 

Orther than Ng, CSF synaptosomal-associated protein-25 (SNAP-25), in particular its soluble form SNAP-25aa40, has shown good accuracy in discriminating AD from HCs and non-AD dementias [79,80,81,82]. Similarly, synaptotagmin-1 and growth-associated protein 43 (GAP 43) CSF concentrations have been shown to be increased in AD-disorders, thus representing a potential biomarker of synaptic degeneration in AD [79,83]. The biomarkers of synaptic dysfunction represent a promising tool for the biomarker-based diagnosis of NDDs; nevertheless, the possible contexts of use of CSF Ng and of the other emerging biomarkers in NDDs still deserve further exploration.

**Table 1:** Overview on the diagnostic/prognostic values and putative contexts-of-use of novel fluid biomarkers for NDDs.

Reference	Protein	Study Population	Diagnostic Value	Prognostic Value	Application
Blood Matrix					
Tau Pathology					
Janelidze S. et al., 2020 [35]	p-tau181	Cohort 1: *n* = 182 (MCI *n* = 28, AD *n* = 38, non-AD dementia *n* = 52, CU *n* = 64) Cohort 2: *n* = 344 (MCI *n* = 125, CU *n* = 219) Cohort 3 (neuropathology cohort): *n* = 63 (AD *n* = 16, non-AD dementia *n* = 47)	p-tau181 differentiating: 1. Cohort 1: - tau-positive vs. negative AuROC = 0.87 with tau-PET as reference; - AD vs. non-AD dementia: AuROC = 0.94 with clinical diagnosis 2. Cohort 1 and cohort 2 together: - Aβ + vs. Aβ-participants: AuROC = 0.80 with Aβ PET as reference 3. Cohort 3: - AD vs. non-AD dementia: AuROC = 0.85 neuropathological reference	Cohort 2: higher p-tau levels are associated with progression to AD for both CU (HR = 2.48) and MCI (HR = 3.07)	Feasible for screening and early diagnosis of AD, with p-tau231 displaying the earliest increase during the disease course
Thijssen E. et al., 2020 [36]		Cohort 1: *n* = 362 (MCI *n* = 47, AD *n* = 56, CBS *n* = 39, PSP *n* = 48, bvFTD *n* = 50, nfvPPA *n* = 27, svPPA *n* = 26, CU *n* = 69) Cohort 2: *n* = 42 (MCI, AD)	p-tau181 differentiating: 1. Cohort 1 - AD vs. FTLD (*n* = 190): AuROC = 0.89 with clinical diagnosis as reference; - Aβ-PET + vs.-: AuROC = 0.86 with amyloid-PET as reference; - autopsy-confirmed AD (*n* = 15) vs. FTLD-tau (*n* = 52) AuROC = 0.86 with neuropathology as reference	NA	
Karikari T. et al., 2020 [37]		*n* = 1331 Discovery cohort: *n* = 37 (AD *n* = 19, CU *n* = 18) Validation cohort 1: *n* = 763 (MCI *n* = 45, AD *n* = 33, FTD *n* = 8, CU *n* = 140) Validation cohort 2: *n* = 763 (MCI *n* = 191, AD *n* = 126, bvFTD/PPA *n* = 18, PD/MSA *n* = 36, VD *n* = 12, PSP/CBS *n* = 21, CU *n* = 337) Primary care cohort: *n* = 105 (MCI *n* = 12, AD *n* = 10, CU *n* = 83)	p-tau181 differentiating: 1. Across cohorts AD vs. CU: AuROCs = 0.90–0.98 2. Validation cohort 1 AD vs. FTD: AuROCs = 0.76–0.82 with clinical diagnosis as reference ● Across cohorts tau-PET + vs. tau-PET-: AuROCs = 0.83–0.93 with tau-PET as reference; AD vs. Aβ-: AuROC = 0.99 with amyloid-PET as reference ● Validation cohort 2 - AD vs. VD: AuROC = 0.92 - AD vs. PSP/CBS: AuROC = 0.89 - AD vs. PD/MSA: AuROC = 0.82 with clinical diagnosis as reference ● Primary care cohort - AD vs. CU young adults: AuROC = 1.0 - AD vs. CU older adults: AuROC = 0.84 with clinical diagnosis as reference	NA	NA
Palmqvist S. et al., 2020 [41]	p-tau217	Cohort 1 (neuropathology cohort): *n* = 81 (AD *n* = 34, non-AD *n* = 47) Cohort 2: *n* = 699 (MCI *n* = 178, AD dementia *n* = 121, non-AD dementia: PD/PDD/MSA, PSP/CBS, bvFTD/PPA, VD *n* = 99, CU *n* = 301) - Cohort 3: *n* = 622 PSEN1 mutation carriers *n* = 365; age- and sex-matched noncarriers: *n* = 257	p-tau181 differentiating: 1. Cohort 1 AD vs. non-AD: AuROC = 0.89 with neuropathology as reference 2. Cohort 2 - AD vs. non-AD dementia: AuROC = 0.96 with clinical diagnosis as reference - tau + vs. tau-: AuROC = 0.93 with tau-PET as reference- amyloid + vs. amyloid -: AuROC = 0.87 with amyloid-PET as reference	NA	NA
Barthélemy NR. et al., 2020 [40]		Discovery cohort: *n* = 36 (non-AD MCI *n* = 2, preclinical AD *n* = 5, AD-MCI *n* = 8, moderate AD *n* = 4, CU *n* = 17) Validation cohort: *n* = 92 (non-AD MCI *n* = 11, preclinical AD *n* = 20, AD-MCI *n* = 24, moderate AD *n* = 6, CU *n* = 31)	p-tau181 differentiating amyloid + vs. amyloid- - discovery cohort: AuROC = 0.98 - validation cohort: AuROC = 0.75 with amyloid-PET or CSF Aβ1-42/Aβ1-40 as reference; p-tau217 differentiating amyloid + vs. amyloid- - discovery cohort: AuROC = 0.99; - validation cohort: AuROC = 0.92 with amyloid-PET or CSF Aβ1-42/Aβ1-40 as reference	NA	NA
Ashton NJ. et al., 2021 [43]	p-tau231	Cohort 1 *n* = 48 (AD *n* = 20, CU *n* = 18) Cohort 2 *n* = 313 (AD *n* = 42, MCI *n* = 54, non-AD dementia *n* = 26, CU *n* = 191) Cohort 3 *n* = 190 (AD *n* = 34, MCI *n* = 17, CU *n* = 139) Cohort 4 (neuropathology cohort) *n* = 47 (AD *n* = 36, non-AD *n* = 11)	p-tau231 differentiating: - AD vs. amyloid-PET- CU: AuROCs = 0.92–0.94 - AD vs. non-AD NDDs (FTD, PSP, CBS, VD, HS, CAA) AuROC = 0.93 - AD vs. amyloid-PET-MCI: AuROC = 0.89 with clinical diagnosis as reference - AD vs. non-AD pathology: AuROC = 0.99 with neuropathology as reference	NA	NA
Mattsson N. et al., 2017 [50]	NfL	*n* = 570 (AD *n* = 180, MCI *n* = 197, CU *n* = 193)	NfL discriminating AD vs. CU participants: AuROC = 0.87 with clinical diagnosis as reference	NA	Potentially suitable to rule out neurodegeneration across different NDDs in primary care settings, eligible for diagnostic and prognostic purposes in AD and ALS
Lewczuk P. et al., 2018 [45]		*n* = 99 (AD *n* = 33, MCI *n* = 25, CU *n* = 41)	NfL differentiating → - diseased participants vs. CU: AuROC = 0.85 - AD vs. CU: AuROC = 0.92 with clinical diagnosis as reference	NA	
Gille B. et al., 2019 [54]		*n* = 250 (ALS *n* = 149, ALS mimics *n* = 19, disease controls *n* = 82)	NfL discriminating ALS vs. ALS mimics: AuROC = 0.85 with clinical diagnosis as reference	Nfl levels in the prediction of mortality among ALS patients: -upper tertile: HR = 5.34 -middle tertile: HR = 4.47	
Verde F. et al., 2019 [57]		*n* = 283 (ALS *n* = 124, FTD *n* = 20, AD *n* = 20, PD *n* = 19, CJD *n* = 6, disease controls *n* = 44, non-NDDs controls *n* = 50)	Nfl differentiating ALS vs. - non-NDDs controls: AuROC = 0.97; - disease controls: AuROC = 0.87; - all other categories together: AuROC = 0.88 with clinical diagnosis as reference	In the prediction of mortality: Nfl levels above the median (125 pg/mL): HR = 2.39	
Thouvenot E. et al., 2020 [58]		*n* = 219 (ALS *n* = 198, CU *n* = 21)	Nfl differentiating ALS vs. CU: AuROC = 0.99 with clinical diagnosis as reference	In the prediction of mortality: Nfl levels ≥ 71,2 pg/mL HR = 4.7	
Mattsson N. et al., 2016 [73]	Ng	*n* = 389 (AD *n* = 93, MCI *n* = 187, CU *n* = 109)	Ng differentiating AD vs. CU: AuROC = 0.71 with clinical diagnosis as reference	NA	Candidate supportive biomarker in the diagnostic/prognostic work-up of AD
Tarawneh R. et al., 2016 [72]		*n* = 302 (AD *n* = 95, CU *n* = 207)	In the discrimination of AD from CU → - Ng: AuROC = 0.71 - Ng + Aβ1-42: AuROC = 0.81 with clinical diagnosis as reference	In predicting the conversion from HC to AD → -Ng: adjusted HR = 1.89 - Ng/Aβ1-42: adjusted HR = 27.9	
Blennow K. et al., 2019 [71]		*n* = 191 (AD *n* = 46, CJD *n* = 81, CU *n* = 64)	Ng differentiating → - CJD vs. CU: AuROC = 0.96 - AD vs. CJD: AuROC = 0.85 - AD vs. CU: AuROC = 0.73 with clinical diagnosis as reference	NA	

Abbreviations: AD: Alzheimer′s disease; AuROC: area under the receiver operating curve; ALS: amyotrophic lateral sclerosis; Aβ: amyloid β; Aβ_1-40_: amyloid β-peptide 1-40; Aβ_1-42_: amyloid β-peptide 1-42; bvFTD: behavioural variant frontotemporal dementia; CBS: corticobasal syndrome; CJD: Creutzfeldt–Jakob disease; CSF: cerebrospinal fluid; CU: cognitively unimpaired; FTD: frontotemporal dementia; FTLD; frontotemporal lobar degeneration; HR: hazard ratio; MCI: mild cognitive impairment; MSA: multiple system atrophy; NA: not assessed; NfL: neurofilament light chain; nfvPPA: nonfluent variant primary progressive aphasia; NDD: neurodegenerative disease; Ng: neurogranin; PD: Parkinson′s disease; PDD: Parkinson′s disease dementia; PPA: primary progressive aphasia; PSP: progressive supranuclear palsy; p-tau181: phospho-tau181; p-tau217: phospho-tau217; p-tau231: phospho-tau231; SMC: subjective memory complainers; svPPA: semantic variant primary progressive aphasia; VD: vascular dementia.

#### 3.2.2. CSF YKL-40

YKL-40 protein measured in CSF is emerging as a reliable indicator of neuroinflammation, a pathophysiological pathway common to the whole NDDs spectrum. CSF YKL-40 showed only moderate accuracy in the discrimination of AD from both HC and non-AD dementias (e.g., DLB and FTD) and in tracking the clinical progression of MCI to full-blown dementia due to AD [84,85]. Several autoptic studies report an association between CSF YKL-40 and brain tau deposits across different tauopathies [86,87,88], suggesting a link of YLK-40 and tau-driven neurodegenerative mechanisms. Additionally, some data show higher levels of CSF YKL-40 in ALS subjects compared to HC [89,90]. Further studies are needed to better elucidate the role of CSF YKL-40 as a peripheral surrogate of neurodegeneration-related mechanisms in the pre-symptomatic/early stages of NDDs and its role as a monitoring biomarker for targeted anti-inflammatory therapies.

## 4. Alternative Matrices

Recently, the research for biomarkers derived from body fluids other than CSF and plasma has raised a great deal of interest. The object is to find the successful, less invasive combination of biomarkers, able to play a role in early diagnosis of NDD as a first-level screening, or to stratify high-risk populations using novel tools as protein misfolding amplification assays. 

### 4.1. Saliva

The well-known markers of amyloid cascade and axonal damage have been measured on saliva of AD patients, providing significant results, although not yet useful as clinical biomarkers. An analysis of salivary p-tau/t-tau ratio levels revealed a significant difference between AD subjects and HCs at S396 phosphorylation site, but with limited discriminatory accuracy [91]. Similar investigations were carried out analyzing salivary α-synuclein concentration in PD patients. Salivary total α-synuclein levels reported an excellent specificity but poor sensitivity to discriminate PD from HCs, while sensitivity increased for oligomeric α-synuclein alone or α-syn_olig_/α-syn_total_ ratio [92]. Finally, the neuroprotective mediator heme oxygenase-1 measured in saliva reported a significantly higher concentration in early PD patients than control subjects [93].

### 4.2. Olfactory Mucosa

The olfactory epithelium (OE) is recently gaining attention in NDDs, potentially reflecting the pathological alterations within the brain. Tau, Aβ, and α-syn have been detected in the OE in patients with NDDs, but frequency, extent, and disease specificity of misfolded protein expression in olfactory mucosa is not yet determined [94]. In AD, neurological dysfunction in central and peripheral olfactory systems has been described. Previous studies found the spreading of soluble Aβ aggregates from peripheral to central olfactory centers even earlier than spatial memory dysfunction [95]. Recently, BSC4090 (a fluorescent ligand targeting neurofibrillary tangles) proved to be an interesting candidate proxy of AD pathology modification in olfactory mucosa biopsies. It fairly distinguished AD from HC (AUROC 0.778) with good accuracy [96] (Table 2). RT-QuIC analyses for detection of α-syn in the olfactory mucosa of PD patients is reliable in both PD and prodromal PD patients [97], and in DLB and prodromal DLB patients too [98] (Table 2). According to a recent study, RT-QuIC resulted positive for α-syn deposition in the olfactory mucosa in 44.4% of REM sleep behavior disorder (RBD) patients [99] (Table 2).

### 4.3. Skin

Skin biopsies have been recently gaining attention for their potential diagnostic role in synucleinopathies. Deposits of p-α-syn have been reported in skin nerve fibers of PD patients but not in atypical parkinsonisms without synucleinopathy. Analysis of p-α-syn deposits in cutaneous nerve fibers are potential tools to predict phenoconversion in RBD patients. According to recent studies, RBD patients positive for p-α-syn depositions in skin biopsies progressed into PD or DLB during a 3-year-follow-up [100] (Table 3). Interestingly, the percentage of p-α-syn-positive fibers in skin biopsies in RBD patients was associated with the probability to be diagnosed as prodromal PD [101] (Table 3). Finally, RT-QuIC techniques proved 95% sensitivity and 100% specificity in detecting α-syn on skin biopsies in PD patients [102] (Table 3).

### 4.4. Urine

Traditional core biomarkers still remain poorly explored in urine. In AD, urinary concentration of exosomes-derived Aβ_1-42_ might differentiate AD patients from HC [103], but results are only preliminary. Separately, a large variety of metabolites demonstrated excellent discrimination between MCI and HC (for isoleucine, acetate, trimethylamine n-oxide, kynurenine, C2, SDMA, malonate, and 5-aminopentanoate), MCI and AD (for glucose, guanidinoacetate, urocanate, hippuric acid, cytosine, 2- and 3-hydroxyisovalerate, 2-ketoisovalerate, tryptophan, and malonate), AD and HC (for 2-hydroxyisovalerate, acetate, ethanolamine, pyridoxine, 2-hydroxybutyrate, and alpha-ketoisovalerate differentiating), and thus suggesting a possible utility for developing a test based on urine metabolomics in this regard [104].

The α-syn concentration in urine was assessed in PD patients with traditional techniques, resulting in different oligomeric forms of α-syn between PD and HCs Fare clic o toccare qui per immettere il testo [105]. Lastly, further urinary metabolite, neopterin, differentiated ASL and HC with similar accuracy, and elevated concentration was associated to longer survival, but little worthwhile evidence is available [106].

### 4.5. Retina

The retina has recently gained attention as a new source of potentially promising biomarkers. The thinning of the retinal ganglion cell inner plexiform layer has been associated with the gray matter volume obtained from MRI scans, suggesting that the retinal ganglion cells are potential markers of cerebral neurodegeneration [107]. In AD, elevated Aβ_42/40_ peptides and p-tau in the retina have been reported [108], with linear correlation between retinal and brain Aβ concentration [109]. Neuronal degeneration, retinal thinning, vascular abnormalities, and gliosis were detected in AD patients [110]. In regards to PD, decreased retinal vessel density and perfusion density were detected, as well as choroidal structural changes in PD patients [111].

## 5. Protein Misfolding Amplification Assays for the In Vivo Dissection of NDDs and for the Stratification of at High-Risk Populations

### 5.1. Protein Misfolding Amplification Assays: An Overview

Originally developed for the in vivo detection of the prion protein, seeding amplifications assays are currently regarded as a reliable diagnostic tool for the diagnosis of prion disorders [112]. Recently, a growing body of evidence supports the role of these techniques for the in vivo assessment of protein misfolding and abnormal accumulation in NDDs [113,114].

**Figure 1:**
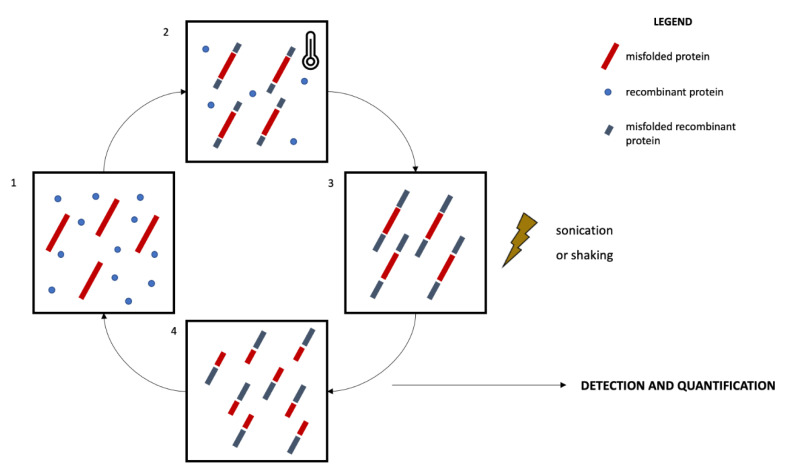
Schematic representation of the protein misfolding amplification assays. The recombinant protein is incubated with the biological sample containing an amount of the misfolded protein to be quantified for a defined time and at a specific temperature (1, 2). This process promotes the further misfolding of recombinant proteins and the elongation of the fibrils (nucleation, 3). The sample then undergoes a sonication (PMCA) or shaking (RT-QuIC) process promoting the fragmentation of the fibrils (4) and the amplification of seeds promoting further misfolding and amplification. The incubation-sonication/shaking cycles (2, 3, 4) are repeated several times, enabling the amplification of small amounts of misfolded proteins. The quantification process occurs real-time in RT-QuIC by means of a fluorescent dye, while it occurs at the end of the incubation-sonication cycles in PMCA by means of Western blotting protocols.

Two main protein misfolding amplification assays have been developed so far, namely the RT-QuIC and the PMCA [115] (Figure 1). Both techniques enable the detection of small amounts of misfolded proteins by exploiting the ability of these species to further seed the misfolding of natively unfolded proteins [116]. The alternating cycles of nucleation, during which the misfolded species are amplified and aggregate into fibrils, and fragmentation in fact make quantifiable even small amounts of misfolded proteins [113,116]. The main technical differences between RT-QuIC and PMCA are related to the fragmentation and quantification systems. In RT-QuIC, the fragmentation is obtained by mechanical shaking. The detection and quantification of the misfolded proteins occur in real-time thanks to a fluorescent dye, which emits a signal when interacting with fibrils, thus reflecting their concentration [117]. In PMCA, the fragmentation is obtained by means of a sonication process and the quantification of the reaction products does not take place real-time during the reaction, occurring instead at the end of the amplification phase. Traditional immunoassays like Western blotting are in fact coupled to the PMCA reaction to enable the quantification of misfolded species [113,117]. Standardization of pre-analytical and analytical issues are still missing. Nevertheless, the high sensitivity and accuracy of protein amplification assays in detecting misfolded species, considered pivotal in NDDs, are promising for an in vivo biological definition of NDDs.

### 5.2. Protein Misfolding Amplification Assays in NDDs: Promising Tools for Disease Stratification

The most robust evidence regarding the potential utility of protein misfolding amplification assays in NDDs comes from the field of α-synucleinopathies [113].

Misfolded α-syn can in fact be detected by means of RT-QuIC in the CSF of patients with α-synucleinopathies, enabling the differentiation of this group of disorders from non-α-synuclein-related mimics [118,119,120,121]. CSF α-syn PMCA is also reported to properly differentiate patients with α-synucleinopathies from other neurological conditions [122,123]. Both assays seem to be potentially useful in the stratification of the spectrum of α-synucleinopathies, being able to differentiate Lewy-bodies-related disorders (LBD), namely PD and DLB, from other α-synucleinopathies like MSA [121,123,124]. Although a direct cross-platform comparison between RT-QuIC and PMCA is missing, preliminary data support a selective accuracy of RT-QuIC in detecting LBD and of PMCA in identifying MSA, thus supporting the notion that different α-synuclein strains may underly these disorders [125]. Intriguingly CSF protein misfolding amplification assays seem to represent potential tools for the stratification of prodromal α-synucleinopathies and for the screening of at high-risk patients. These include patients with RBD [121,126,127] (Table 4), with PAF [121], and carriers of mutations associated with genetic forms of PD (e.g., LRRK2 and GBA) [121,128]. Preliminary results from longitudinal studies support the notion of an increased risk of phenoconversion to overt α-synucleinopathy in CSF α-syn-RT-QuIC-positive patients compared to negative ones [127]. More recently, RT-QuIC was able to detect α-synuclein in the olfactory mucosa of patients with PD and RBD [97,99] (Table 4), opening new scenarios for a minimally invasive screening of patients with full-blown and prodromal α-synucleinopathies. The use of protein amplification assays for the detection of other misfolded proteins in CSF is still in its infancy. Nonetheless, preliminary results support a utility of CSF RT-QuIC in detecting 3R tau aggregates in Pick′s Disease (PiD) [129], 4R tau aggregates in PSP and CBD [130], and TDP-43 inclusions in conditions belonging to the ALS-FTD spectrum [131]. As regards TDP-43, RT-QuIC was able to detect this protein also in pre-symptomatic mutation carriers [131]. At last, a single report suggested that CSF Aβ_1-42_ PMCA assay could differentiate AD from non-AD neurological disorders [132]. To sum up, ultrasensitive protein amplification assays are now regarded as potential tools for the stratification of NDDs from their prodromal to their full-blown stages. They may open a window on key pathophysiological mechanisms and enable the in vivo tracking of dynamic time-trajectories of early neurodegenerative changes in NDDs. Although promising, these results deserve a more extensive validation and standardization, cross-platform comparisons, and further prospective evaluations.

## 6. Biomarkers in Clinical Trials for NDDs

Clinical trial protocols for DMT of NDDs typically employ CSF fluid biomarkers as inclusion criteria or measure of study outcomes, and plasma biomarker as a measure of study outcomes. However, novel trials increasingly rely on plasma biomarkers, especially those investigating novel strategies. Here, we collected the most relevant information about biomarkers and clinical trials for AD and genetic forms of NDDs from Clinicaltrial.gov, updated at 31 May 2021.

### 6.1. AD

The current role of biomarkers in AD clinical trial is to ensure appropriate patient selection, intercepting subjects in the Alzheimer′s continuum [9]. The demonstration of targeted engagement seems to be crucial; however, less than 60% of current trials relies on it [133]. On the other hand, each of the proposed trials enrolled patients with evidence of amyloid pathology (PET or CSF detection) in different clinical stages of disease (prodromal, early symptomatic, mild AD). Indeed, core AD biomarkers are clinically established in tertiary centers for diagnosis and trial enrollment.

DMT strategies are mainly focused on amyloid or tau pathology, as the avoidance or clearance of misfolded protein deposition are still the most studied pharmacological approaches in AD and other NDDs. Furthermore, neuroinflammation is an emergent pathophysiological mechanism with possible alternatives of treatment compared to classical proteinopathy-based strategies. 

#### 6.1.1. Anti-Amyloid Strategies

The target of most DMT for AD remains to be amyloid pathology, despite the failure of many trials with anti-amyloid strategies.

Anti-amyloid engaged drugs are designed according to two main mechanisms: interference upstream of Aβ peptide generation, by enzymatic inhibition of γ-secretase or BACE1, which physiologically cleave APP into beta-amyloid peptides, or downstream of Aβ peptide generation, by promoting its removal in either mono/oligomeric, aggregated, or plaque form via active or passive immunization [134].

None of the BACE or gamma-secretase inhibitors successfully concluded phase III trials, either due to the lack of significant clinical effect or an unfavorable benefit/risk profile [135,136]. Among the “survivor” anti–β-amyloid drugs, there is one vaccine (ABvac40) and six monoclonal antibodies (Gantenerumab, Solanezumab, Lecanemab [BAN2401], Aducanumab, Donanemab, Crenezumab) now under phase II or III of clinical trials. 

The ABvac40 phase I study did not consider CSF biomarkers as outcome measures. However, ABvac40 active immunization was measured with the presence of anti-Aβ_1-40_ antibodies and plasma Aβ_1-40_ levels [137]. The results from the phase II trial are still unavailable, nor the interim analysis presented at AAIC 2020 congress [138,139].

Gantenerumab, a fully human IgG1 antibody that binds a conformational epitope on Aβ fibrils, was initially tested at low-dose in two phase III studies, the SCarlet RoAD and Marguerite RoAD studies, and was able to significantly reduce in dose-dependent manner CSF p-tau and t-tau levels in prodromal AD patients [140]. This finding supported two trials (Graduate 1 and Graduate 2) currently active that consider CSF tau/Aβ_1-42_ ratio as inclusion criteria; regarding established study outcome on CSF Aβ_1-42_, p-tau, t-tau, there are no results available.

Solanezumab is a humanized monoclonal IgG1 antibody that recognizes Aβ peptide just in the soluble monomeric form, and inhibits Aβ aggregation [141]. In trials designed for prodromal to moderate AD (EXPEDITION 1, 2, 3, PRO), CSF and plasma Aβ_1-40_ and Aβ_1-42_ biomarkers were used as outcome measures and the drug proved to be effective in target engagement with increased concentrations of both [141,142,143]. Though these trials were either terminated early or had failed clinical and functional endpoints, biomarker outcomes suggested the drug to be tested in cognitively healthy older adults with evidence of amyloid pathology (A4 study). In the ongoing trial (no results available), liquid biomarkers are among secondary study outcomes, as changes in CSF Aβ_1-42_, p-tau, t-tau levels from baseline [144].

Lecanemab (BAN2401) is a humanized IgG1 monoclonal antibody that preferentially binds to soluble Aβ oligomers and protofibrils [145]. CSF Aβ_1-42_, p-tau, Ng, and NfL were evaluated as study outcomes as evident from recently published results of phase 2b study. The group treated with lecanemab 10 mg/kg monthly and 10 mg/kg biweekly, showed a substantial normalization of core AD and closely related biomarkers, with the increase in CSF Aβ_1-42_, reduction in CSF p-tau levels, and a trend regarding the reduction of CSF Ng and NfL [146].

Finally, aducanumab, a fully human IgG1 monoclonal antibody that binds aggregated forms of Aβ [147], received accelerated approval by the FDA on 7th June 2021. Only limited information is available about the results of the ENGAGE and EMERGE studies, two twin phase 3 studies on MCI and mild dementia due to AD. Evaluation of biomarker data shows a significant reduction in CSF p-tau but not t-tau levels in the treatment group [148]. An interim analysis of aducanumab clinical studies led to early termination for futility in March 2019, was redeemed after further post-hoc analysis with consequent FDA approval, and now is under open label phase III trial (EMBARK).

AD monogenic forms deserve a separate discussion. Solanezumab and gantenerumab were tested in a phase 2/3 trial for dementia prevention in pre-symptomatic and mildly symptomatic PSEN1/PSEN2/APP mutation carriers within the Dominantly Inherited Alzheimer Network Trials Unit (DIAN-TU) [149], considering the DIAN Multivariate Cognitive Endpoint and fluid biomarkers as outcomes. Both drugs′ dosing were augmented four-to-five-fold during the trial due to evidence coming from trials on sporadic AD subjects. Results were presented at the AAIC 2020 conference. Solanezumab increased CSF Aβ_1-42_, but also NfL. Furthermore, the treatment group showed an accelerated decline in cognition to placebo [150]. Clinical worsening agreed with the increase of NfL as a liquid biomarker of neuronal degeneration. Despite showing a significant reduction of CSF t-tau, p-tau181, and NfL, Gantenerumab did not have the expected positive effect on cognition [151].

#### 6.1.2. Anti-tau Strategies

Regarding tau pathology, therapeutic approaches comprise (a) active and passive immunization, (b) kinase inhibitors, meant to interfere with tau phosphorylation, (c) tau aggregation inhibitors, and (d) microtubule-stabilizing agents, that compensate p-tau loss of function [152]. Among the drugs now under phase II or III trials, there are two vaccines (AADvac1, ACI-35), five monoclonal antibodies (Semorinemab, Tilavonemab, Gosuranemab, Zagotenemab, and JNJ-63733657) and one aggregation inhibitor (TRx0237).

AADvac1 uses an epitope from amino acids 294 to 305 of the tau sequence, coupled to keyhole limpet hemocyanin [153]. CSF core AD biomarkers were optional as inclusion criteria for the phase II trial ADAMANT on mild AD, concluded in June 2019. The CFS levels of p-tau181 and p-tau217 and NfL were evaluated during the trial and the results were announced in a press release (9 September 2019) AADvac1 showed efficacy in inducing immunization, plus a significant slow-down of the expected increase of NfL in treated patients, and a trend in reduction of p-tau in the CSF [154]. Despite the promising results, no phase III trial has been announced yet.

ACI-35 is a vaccine directed against a synthetic tau fragment phosphorylated at sites pS396/pS404, with an improved version (ACI-35.030) currently in phase II clinical trial in MCI due to AD or Mild AD subjects. However, no fluid biomarker is included in the protocol as outcome measure [155].

Semorinemab, a monoclonal antibody that targets all six isoforms of tau at the N-terminus, both monomeric and oligomeric, phosphorylated or not, was tested in two phase II trials on prodromal to mild AD (TAURIET, currently completed) and moderate AD (LAURIET, currently active, not recruiting). Both trials considered core AD and neuroinflammatory/neurodegenerative liquid biomarkers as outcome measures. Despite failing clinical and functional endpoints, during TAURIET, semorinemab reported lowered CSF t-tau and p-tau levels and increased tau in plasma, moreover it was influential on other CSF biomarkers like NfL, Ng, S100B, and sTREM1, as presented at International Conference on Alzheimer′s and Parkinson′s Diseases 2021 (unpublished results) [156].

Other antibodies in phase II study do not consider liquid biomarkers in their protocol after patient inclusion except JNJ-63733657. For this antibody, CSF concentrations of total, free, and bound p217 tau fragments were measured. However, no results are available so far, except for the reduction in CSF free p217 tau of healthy subjects from a previous phase I study [157].

#### 6.1.3. Neuroinflammation

Following epidemiological studies that reported a lower incidence of AD in NSAIDs long-term users [158], an alleged role in prevention was given to NSAIDs, but prospective studies disproved this hypothesis [159,160]. Nevertheless, inflammation is still believed to be one of the major factors implied in the AD pathophysiological process, and more targeted drugs are under development against it. Neuroinflammation is particularly oriented on the role of neuron-glial cell (both astrocytes and microglia) interactions, neural extracellular matrix (nECM), and signaling from the neurovascular unit [161]. Among those with the most definite target engagement, there are two monoclonal antibodies, AL002 and pepinemab.

AL002 binds the microglial receptor TREM2 and activates its signaling, thus modulating Aβ pathology through microglial scavenging [162,163]. However, the phase II trial (INVOKE-2) does not use specified CSF and plasma biomarkers other than for enrollment [164].

Pepinemab is a monoclonal antibody directed against semaphorin 4D, a glycoprotein released by neurons in response to stress, that binds a receptor on astrocytes and oligodendrocytes in CNS [165], claimed to be upregulated in AD and HD subjects [166]. A phase II trial is now running with MCI to mild AD with serum and CSF neuroinflammatory cytokines dosage (IL-1, IL-2, IL-4, IL-5, IL-6, IL-10, IL-12, IL13, IFNγ, TNF-α, TGFβ), T- and B-lymphocytes’ quantitation, plasma and CSF NfL, plasma and CSF Aβ_1-42_/Aβ_1-40_, CSF t-tau and p-tau, CSF YKL-40 concentrations as secondary outcomes (results not available) [167,168].

### 6.2. Genetic Forms of NDDs

#### 6.2.1. FTD

FTD is a heterogeneous NDDs, clinically presenting with behavioral disturbances or language disorders. Moreover, an overlap with ALS or parkinsonism (generally PSP or CBS) may occur [169]. ALS and FTD present a considerable clinical, pathological, and genetic overlap, and are currently considered as opposite poles of the same disease spectrum [170]. The TDP-43 accumulation is the pathological hallmark of ALS, whereas FTD patients show either TDP-43 or tau proteinopathy with a smaller number of cases with FUS pathology.

Genetic forms account for nearly 30% of FTD cases [169]. Three genes are mostly involved in autosomal dominant cases of FTD: progranulin (GRN), microtubule-associated protein tau (MAPT), and chromosome 9 open reading frame 72 (C9orf72) [169]. MAPT mutations generally exhibit full penetrance, while GRN and C9orf72 show age-related penetrance [171,172]. Different genotypes have been associated with different clinical signs [173] and brain imaging patterns.

The identification of genetic mutations in FTD patients is paramount for the development of personalized treatments as well as the discovery of specific biomarkers. 

Three biomarkers have recently gained attention: NfL, progranulin, and poly(G) dipeptide repeat proteins (DPRs) [169]. NfL levels reflect axonal damage is not specific for FTD but could be used as outcome measure for successful treatments in clinical trials [174]. Low concentration of progranulin in CSF or plasma is reported in GRN-mutated FTD, while C9orf72 mutation carriers show increased CSF poly(G) DPRs [169].

Three new drugs are currently under investigation in phase II trials for GRN mutation carriers. AL001 is a recombinant human anti-human monoclonal IgG1 targeting sortilin (SORT1), which serves as a lysosomal trafficking receptor for progranulin. Results of phase II (INFRONT-2) and phase III (INFRONT-3) trials are not yet available. Data from phase I trial showed a dose-dependent increase of plasma and CSF progranulin levels in both symptomatic and asymptomatic FTD patients [175]. Change in blood-based biomarkers and optional CSF biomarkers (NfL and progranulin) will be determined in INFRONT-3 trial [176].

Another therapeutic approach is based on gene therapy. PBFT02 and PR006 are adeno-associated viral vectors (AVV) carrying GRN, the gene encoding for human progranulin to patients′ cells. No data on biomarkers are available for PBFT02 [177]. The phase 2 trial PROCLAIM testing PR006 is currently ongoing and blood and CSF concentration of progranulin and NfL will be assessed. No preliminary results are still available [178].

TDP-43 is present in inclusion in nearly 50% of FTD patients [179] characterized by hyperphosphorylation at Serine 409/410. Indeed, the protein casein-kinase 1δ (CK-1δ) inhibitors are promising targets, but no specific human studies or biomarkers have been proposed [180].

#### 6.2.2. ALS

ALS is characterized by high variability both in phenotype and genetic background. Around 10% of ALS patients have genetic mutations [181]. The most common are C9orf72 (40%), SOD1 (20%), FUS 1–5%, and TARBDP (1–5%) [182]. Abnormal aggregation of TDP-43 is the major pathological background (~95% of cases), followed by accumulation of misfolded SOD1 and FUS [183].

The identification of reliable biomarkers with clinical utility in pharmacological trials is hampered by the multiple pathophysiological mechanisms underpinning ALS [184]. However, in genetic ALS, due to C9orf72 and SOD1 mutation carriers, poly(G) DPRs and SOD1 proteins assessed in CSF are encouraging. Importantly, NfL measured either in plasma or CSF is a promising fluid biomarker in ALS, reflecting the rate of disease progression and the involvement of upper motor neuron [184]. Thus, it could be used as surrogate of neurodegeneration progression in clinical trials. 

Two antisense oligonucleotides are currently under investigation in genetic forms of ALS. BIIB067 (Tofersen) is an antisense drug that binds specifically to SOD1 mRNA, stopping SOD1 protein production [185]. Phase 3 trial is currently ongoing, and changes in CSF SOD1 concentration will be also evaluated. On the other hand, C9orf72 is targeted by BIIB078 in an ongoing Phase 1 trial [186], but liquid biomarker are not assessed. 

FUS is a DNA/RNA binding protein that can be associated with a variety of NDDs apart from ALS (i.e., FTD or polyglutamine diseases). FUS protein is physiologically located in the nucleus, while mutated proteins localize in the cytoplasm forming abnormal aggregates [187]. Mechanisms of the FUS-induced toxicity are debated, and no studies assessed FUS protein as fluid biomarker. A clinical trial recently started in ALS patients with FUS mutation. ION363 is a form of antisense therapy silencing mRNA coding for FUS protein, thus aiming to reduce its intracellular expression. CSF NfL and FUS concentrations will be measured as secondary outcome [188].

## 7. Discussion

The collected evidence suggests that the field of liquid biomarkers (as a liquid biopsy) is continuously evolving, facing the unmet needs of screening, early diagnosis, stratification, and prognosis of NDDs. The causative or core proteins of NDDs are paired with markers of cellular or synaptic damage and inflammation which can be revealed through blood, CSF, or alternative matrices assays. However, the discussed role of biomarkers is not prominent in many of the latest clinical trials for DMTs, which are more focused on clinical evaluation and neuroimaging, secondarily limiting the investigation of liquid biomarkers to CSF core proteins.

The gap between the development and clinical validation of liquid biomarkers is widened by their exclusion in clinical practice. Screening biomarkers, potentially useful to intercept patients in prodromal stages of NDDs are limited to research settings, not implemented in real-life screening campaigns. As a consequence, the enrollment in clinical trials of early-stage diseases is limited to symptomatic patients, except for the genetic forms. 

Moreover, several technical aspects hinder the inclusion of biomarker assessment in clinical routine. Cross-platform evaluations for the wide-scale implementation of a standardized analyte-specific method, unbiased guidelines to homogenize cut-off values as well as pre- and post-analytical procedures, and the cost-effectiveness appraisal when considering the limited availability of highly innovative platforms (e.g., Simoa) are still unmet needs.

The feasibility of the screening approach is further limited by ethical issues since the NDDs have no effective treatment. A biomarker with a high negative predictive value could medicalize asymptomatic people that with further diagnostic assessments could receive an incurable diagnosis years before its actual onset. On the other hand, the evidence seems to point in the direction of an intervention to treat NDDs in their prodromal/preclinical phase. Preventive healthcare is widely accepted for an infective form of pandemic, as we are facing COVID-19. The NDDs, though representing a mounting issue for the rapidly escalating number of patients, are more difficult to approach with screening campaigns.

The accurate selection of candidate populations is the first step to consider proposing an algorithm for clinical screening, diagnosis, and prognosis. The elderly population seems to be the aim of the screening, considering aging as the major unmodifiable risk factor for NDDs, with the proposed target age of ≥65 years for asymptomatic people [189].

**Figure 2:**
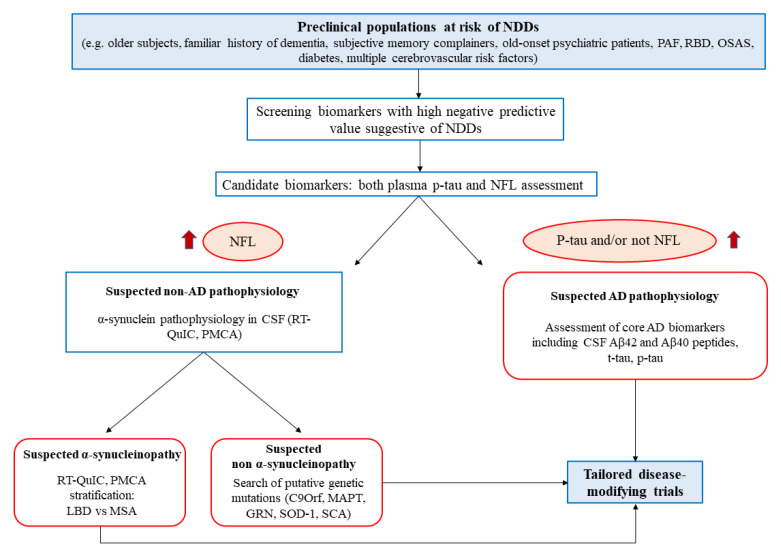
Flowchart representation of the proposed diagnostic algorithm, starting with a high-risk population for NDDs at a preclinical level. The first level of screening is constituted by blood sampling and NFL/p-tau dosage. The screened population will undergo specific diagnostic assessment for suspected AD or be further stratified for α-synucleinopathy through misfolded protein amplification assays. The final goal is to ameliorate the patient quality of life, treat their comorbidities and enroll them into tailored clinical trials. PAF: pure autonomic failure; RBD: rapid eye movement sleep behavior disorder; OSAS: obstructive sleep apnea syndrome; NDDs: neurodegenerative diseases: NFL: neurofilaments RT-QuIC: real-time quaking Induced conversion; PMCA: protein misfolding cyclic amplification; LDB: Lewy body diseases; MSA: multiple system atrophy; CSF: cerebrospinal fluid AD: Alzheimer′s disease.

The suggested age does not maximize the interception of asymptomatic patients, since 1 out of 10 people could have already developed the disease, but it could minimize the ethical issues [189]. Another unmodifiable risk factor to consider is familiarity, which could also increase the willingness of the patient to undergo screening tests, as commonly proposed for the genetic forms. The modifiable risk factors could guide the further selection, considering the metabolic and cardiovascular factors, such as metabolic syndrome, smoke habit, high body mass index in mid-life, type 2 diabetes mellitus, carotid atherosclerosis, hypertension, heart disease, and obstructive sleep apnea [190,191,192]. Following preliminary studies of validation, a clinical score could be made based on these factors to select the candidate population (Figure 2). Moreover, the medicalization of elders with at least two of the presented factors could be useful to treat the comorbidities, ameliorating their general health regardless of the putative NDDs.

A different approach regarding symptomatic patients can be considered, selecting subjective memory complainers [193], patients with RBD [194] or PAF [121], and psychiatric patients presenting atypical/late-onset depression, behavioral disturbances, or psychotic symptoms [61] (Figure 1). For these patients, the age and familiarity factors could be less strict as they would go through differential diagnoses for their disorders. 

Candidate liquid biomarkers for the high-risk populations that can be validated in clinical practice; based on research data, there are blood levels of both NfL and p-tau. These biomarkers have been selected for their high negative predictive values and the feasibility of the sampling. NfL, indeed, lacking specificity, could be used as a screening marker of neuronal damage, being broadly elevated in NDDs also in prodromal stages [44,52,53]. The reported difference in NfL levels, with higher concentrations of plasma protein in PD compared to atypical parkinsonism, should not be taken into account in this phase but eventually evaluated based on the results of the diagnostic algorithm [63,64,65].

The dosage of blood p-tau231 or p-tau181 through either ECLIA-based or Simoa methods could substantially distinguish the NfL positive population, respectively, in non-AD (NfL+, p-tau −) versus AD (NfL +, p-tau +) pathophysiology of the putative NDD [35,43].

The patients selected through these blood biomarkers go through a second level of the diagnostic algorithm. In particular, the AD group should be tested for CSF core proteins (Aβ_1-42_/Aβ_1-40_, p-tau/t-tau), functional imaging (FdG- and amyloid-PET), and diagnosed accordingly to specific criteria [21,195]. In the AD group, the CSF dosage of Ng could be useful to monitor disease progression and distinguish the atypical phenotypes of AD [68,75]. 

The non-AD group can be further studied through the CSF quantification of α-synuclein by means of RT-QuIC and PMCA to differentiate NDDs in α-synucleinopathies and other NDDs. The two novel techniques to measure α-synuclein could be useful also to stratify the α-synucleinopathies in LBD or MSA [121,122]. Eventually, the remaining putative NDDs should undergo conventional diagnostic tests, neuroimaging, and genetic testing (e.g., C9orf72, MAPT, GRN, SOD-1). Progranulin and poly(GP) DPRs CSF levels could be used, respectively, in GRN and C9orf72 mutations to follow disease progression during clinical trials [169].

The alternative matrices for screening or diagnosis or to monitor disease progression are not ready to be applied in the present algorithm; however, RT-QuIC could be applied to skin biopsies to detect α-synuclein in PD patients [102].

The selected patients can be enrolled in specific clinical trials of DMTs, in the early or prodromal stages of the diagnosed disease, potentially ameliorating the drug targeted intervention in phases of the NDDs characterized by higher resilience of the CNS.

Asymptomatic subjects, fully aware and cognitively unimpaired, who decide to undergo this kind of diagnostic work-up should receive adequate counselling before. The diagnosis of a specific pre-symptomatic disease implies the right to know (or to know not), planning, access to DMT trials, but it exposes these subjects to overdiagnosis and overtreatment among the other risks [196]. Further concerns come from those who enter the screening process but fail to meet any of the specific neuropathologies (NfL +, negative all the rest), leaving the subject to deal with an increased risk of a non-further-specified NDD. Future technologies and novel biomarkers implementation will increase the accuracy of screening procedures and progressively reduce the percentage of subjects that fall in this last group. The biomarker of accessible body fluids as blood (first level) and more invasive but easily performable procedures as lumbar puncture for CSF collection (second level), together with emerging matrices, could facilitate the clinical selection of patients. More limited evidence is collected about prognostic and monitoring validity of the available markers. A dosage of liquid biomarker is affordable both in terms of materials and human resources despite some technical drawbacks that are amendable. Moreover, compared to functional and traditional imaging, biomarkers can be reassessed coherently at low cost, are more sensitive to molecular changes, do not require the administration of tracers or contrast enhancers, and could furnish additional information about disease pathophysiology. The proposed algorithm is based on the state-of-the-art research data on liquid biomarkers; more evidence is needed to validate them in clinical practice. Eventually, the flowchart could be improved with emergent neuroinflammatory mechanisms [161,197] and other biomarkers that account for synaptic loss [68] or more specific for primary tauopathies [129,130,131], but further investigations are needed to translate them into clinical applications. Finally, we guess a combined use of multiple biomarkers (Table 5) is mandatory to precisely stratify NDDs depicting the essential characteristic of neurodegeneration such as neurodegeneration tout court (e.g., NFL), specific AD (p-tau and β-amyloid 40–42 peptides), and synuclein-related neurodegenerations (α-syn), and synaptic dysfunction (e.g., Ng). This awaits progress for reliable indicators of neuroinflammation, TDP-43, and 3R/4R pathologies as well.

## Figures and Tables

**Table 2:** Biomarkers in olfactory mucosa in the work-up of NDDs.

Reference	Matrix	Protein	Study Population	Technique	Diagnostic Value	Prognostic Value
Pellkofer et al., 2019 [96]	Olfactory mucosa	BSC4090 (ligand for neurofibrillary tangles), t-tau, p-tau181	*n* = 35 (AD *n* = 12, MCI *n* = 13, HC *n* = 10)	Immunohistochemistry	AD vs. HC: sensitivity 75%, specificity 80%, AUROC 0.778 MCI due to AD vs. MCI non-AD: sensitivity 100%, specificity 71.4%, AUROC 0.857 (AD + MCI due to AD) vs. (HC + MCI non-AD): sensitivity 77.8%, specificity 76.5%, AUROC 0.810	Positive correlation between BSC4090 and p-tau181: *p* = 0.0466, *r* = 0.438846
Perra et al., 2021 [98]	Olfactory mucosa	α-synuclein	*n* = 82 Probable DLB (*n* = 32), prodromal DLB (*n* = 5), mixed dementia (AD/LB *n* = 6), non-α-synucleinopathies (*n* = 38)	RT-QuIC	-DLB vs. non-α-synucleinopathies: sensitivity 81%, specificity 92% -good agreement between clinical diagnoses and RT-QuIC OM testing (k = 0.729) -moderate agreement between RT-QuIC assay in OM and CSF and clinical diagnoses (k = 0.498)	NA
Stefani et al., 2021 [99]	Olfactory mucosa		*n* = 163 iRBD (*n* = 63), PD (*n* = 41), HCs (*n* = 59)	RT-QuIC	iRBD + PD vs. HCS sensitivity 45%, specificity 90%	NA

Abbreviations: AD: Alzheimer′s disease; AuROC: area under the receiver operating curve; CDR: clinical dementia rating; HC: healthy controls; MCI: mild cognitive impairment; p-tau: phosphor tau; t-tau: total tau.

**Table 3:** Biomarkers in olfactory mucosa in the work-up of NDDs.

Reference	Matrix	Protein	Disease	Study Population	Analytical Methods	Diagnostic Value	Prognostic Value
Wang Z. et al., 2020 [102]	Abdominal skin, scalp skin	α-syn	PD, DLB, MSA, AD, PSP, CBD	*n* = 130 Cohort 1: *n* = autopsy sample; PD *n* = 47, DLB *n* = 7, MSA *n* = 3, AD *n* = 17, PSP *n* = 8, CBD *n* = 5, HC *n* = 43 Cohort 2: *n* = 41 biopsy sample; PD *n* = 20, HC *n* = 21	RT-QuIC, PMCA	RT-QuIC on abdominal skin (autopsy): 95% sensitivity and 100% specificity in detecting α-syn in PD vs. HCRT-QuIC on scalp skin (autopsy): 100% sensitivity and 100% specificity in detecting α-syn in PD vs. HC RT-QuIC on abdominal skin (autopsy): PD vs. NNCs AUC 0.9938 RT-QuIC on abdominal skin (autopsy): Synucleinopathies vs. non-synucleinopathies AUC 0.9696 PMCA on skin (autopsy): Synucleinopathies vs. non-synucleinopathies AUC 0.9444 RT-QuIC on skin (autopsy): PD vs. (PSP and CBD) AUC 0.975 PMCA on skin (autopsy): PD vs. (PSP and CBD) AUC 0.913 RT-QuIC on skin (living patients): 95% sensitivity and 100% specificity in detecting α-syn in PD vs. HC, AUC 0.9952 PMCA on skin (living patients): 80% sensitivity and 90% specificity in detecting α-syn in PD vs. HC, AUC 0.9250	NA
Al-Qassabi et al., 2020 [100]	Skin biopsy from cervical area	α-syn	PD, RBD, PSP, vascular parkinsonism, MSA, CBD, drug-induced parkinsonism	Cohort 1 (autopsy): *n* = 51 (DLB *n* = 28, HC *n* = 23) Cohort 2 (living patients): *n* = 79 (RBD, *n* = 28, PD *n* = 20, PSP *n* = 4, vascular parkinsonism *n* = 2, MSA *n* = 1, CBD *n* = 2, drug-induced parkinsonism *n* = 1, HC *n* = 21)	Immunostaining	Skin positivity in 82.1% RBD, 70% PD, 20% atypical parkinsonism, 0% HCs.	At 3-year follow-up, 75% of RBD patients with positive biopsy phenoconverted to defined neurodegenerative disease
Doppler et al., 2017 [101]	Skin biopsy from cervical area, thigh, and leg	p-α-syn	PD, RBD	*n* = 63 (PD *n* = 25, RBD *n* = 18, HC *n* = 20)	Immunofluorescence	p-α-syn in dermal nerve fibres, PD vs. HC: *p* = 0.0001, sensitivity 80%, sensibility 100% p-α-syn in dermal nerve fibres, RBD vs. HC: *p* = 0.0001, sensitivity 55.6%, sensibility 100%	p-α-syn in dermal nerve fibres positively correlated with the total likelihood ratio for RBD to present prodromal PD (ρ = 0.531, *p* = 0.023)

Abbreviations: ACC: accuracy; AD: Alzheimer′s disease; AuROC: area under the receiver operating curve; CIDP: chronic inflammatory demyelinating polyneuropathy; FUS: fused in sarcoma protein; ME: mitochondrial encephalopathy; MG: myasthenia gravis; MS: multiple sclerosis; MSA: multiple system atrophy; NNCs: nonneurodegenerative controls; NPV: negative predictive value; PD: Parkinson′s disease; PD+OH: Parkinson′s disease with orthostatic hypotension; PGRN: progranulin; p-tau: phospho-tau; PMCA: protein misfolding cyclic amplification; PPV: positive predictive value; PSP: progressive supranuclear palsy; RBD: REM sleep behaviour disorder; RT-QuIC: real-time quacking-induced conversion; syn: synuclein.

**Table 4:** Performance of α-synuclein RT-QuIC in populations at higher risk for α-synucleinopathies assessed in CSF and in the olfactory mucosa.

Reference	Study Population	Analytical Method	Matrix	Diagnostic Value	Prognostic Value
Stefani A et al., 2021 [99]	iRBD *n* = 63	RT-QuIC	Olfactory mucosa	iRBD + PD vs. controls: sensitivity 45.2%, specificity 89.8%	NA
Iranzo A et al., 2021 [127]	iRBD *n* = 52	RT-QuIC	CSF	iRBD vs. controls: sensitivity 90.4%, specificity 90%	Risk of developing PD/DLB α-syn- iRBD vs. α-syn+ iRBD: HR = 0.143
Rossi M et al., 2020 [121]	PAF *n* = 18 iRBD *n* = 18	RT-QuIC	CSF	- In iRBD sensitivity 100%, specificity 100%; - In PAF: sensitivity 92.9%	NA
Garrido A et al., 2019 [128]	LRRK2-PD *n* = 15 LRRK2-NMC *n* = 16	RT-QuIC	CSF	- In LRRK2-PD: sensitivity 40% - in LRRK2-NMC: sensitivity 18.8%	NA
Fairfoul G et al., 2016 [126]	RBD *n* = 3	RT-QuIC	CSF	- In iRBD: 100% of patients had positive RT-QuIC	NA

Abbreviations: CSF, cerebrospinal fluid; DLB, dementia with Lewy bodies; HR, hazard ratio; iRBD, idiopatic REM sleep behavior disorder; LRRK2, leucine-rich repeat kinase 2; NMC, non-manifesting carriers; PAF, pure autonomic failure; PD, Parkinson′s disease; RT-QuIC, real-time quaking induced conversion.

**Table 5:** Overview of candidate fluid biomarkers for a stratification of neurodegenerative diseases.

	Matrix	Diagnostic Value			Prognostic Value	Monitoring Treatment
		Preclinical stage	Prodromal stage	Full-blown picture		
*Neurodegeneration*						
Nfl	CSF, Blood	+	+	+	+	±
*AD pathology*						
Aβ peptides	CSF	+	+	+	±	±
p-tau	CSF, Blood	+	+	+	+	±
*Synucleinopathies*						
A-syn prionoids	CSF	+	+	+	±	±
*Synaptic dysfunction*						
Ng	CSF	±	+	+	+	±

Abbreviations: A-syn: α-synuclein; Aβ: β-amyloid; CSF: cerebrospinal fluid; NFL: neurofilament light chain; Ng: neurogranin.

## Data Availability

Not applicable.
